# Oligometastatic Urothelial Cancer and Stereotactic Body Radiotherapy: A Systematic Review and an Updated Insight of Current Evidence and Future Directions

**DOI:** 10.3390/cancers16183201

**Published:** 2024-09-20

**Authors:** Antonio Angrisani, Davide Giovanni Bosetti, Ursula Maria Vogl, Francesco Mosè Castronovo, Thomas Zilli

**Affiliations:** 1 Department of Radiation Oncology, Oncology Institute of Southern Switzerland, EOC, 6500 Bellinzona, Switzerland; davidegiovanni.bosetti@eoc.ch (D.G.B.); francescomose.castronovo@eoc.ch (F.M.C.); 2 Medical Oncology Institute of Southern Switzerland, EOC, 6500 Bellinzona, Switzerland; ursula.vogl@eoc.ch; 3 Facoltà di Scienze Biomediche, Università della Svizzera Italiana, 6900 Lugano, Switzerland; 4 Faculty of Medicine, University of Geneva, 1206 Geneva, Switzerland

**Keywords:** stereotactic body radiation therapy (SBRT), urothelial cancer, oligometastases, metastasis-directed therapy, bladder cancer

## Abstract

**Simple Summary:**

A systematic review was carried out to provide evidence regarding the role of stereotactic body radiation therapy (SBRT) for oligometastatic urothelial cancer (omUC). The present work aims to summarize and critically analyze the available data, with a focus on controversial areas and future directions in this emerging field. Eight studies were identified. Heterogeneous SBRT dose/fractionation and metastatic setting did not allow for a meta-analysis. SBRT showed a good toxicity profile, and the better outcomes for both local control and survival were associated with a biologically effective dose (BED10) ≥ 78 Gy.

**Abstract:**

Background: Stereotactic body radiation therapy (SBRT) is the most commonly used metastasis-directed therapy (MDT) for oligometastatic urothelial carcinoma (omUC). Despite efforts in defining this disease entity, open questions remain concerning the role of MDT and the use of biomarkers, imaging, and its combination with systemic therapies. The aim of the present systematic review is to provide an updated overview of the current clinical evidence on SBRT for omUC in terms of survival and local control benefits. We also aim to provide updates on controversial areas and future directions in this emerging field. Methods: With a systematic approach, following PRISMA recommendations, we searched two databases to identify and select articles published up until March 2024 reporting the use of SBRT for omUC with or without concomitant systemic therapies. Prospective randomized or non-randomized studies as well as retrospective studies were included. Results: Eight studies were selected for data extraction and 293 omUC patients treated with SBRT were collectively analyzed. In metachronous omUC patients, SBRT delivered with ablative doses (BED10 ≥ 78 Gy) was associated with a 2-year overall survival (OS) rate of 50.7% (95% CI 35.1–64.4%). The use of sub-ablative SBRT doses (BED10 = 43.2 Gy) in combination with immunotherapy did not demonstrate significant clinical outcome improvement in two prospective studies. The overall tolerance was good, with only one study reporting toxicity of grade 3 in up to 18% of the patients treated with SBRT in combination with immunotherapy. Conclusions: SBRT is an effective and widely available MDT option in omUC, although this is based on a limited number of studies. Despite the attempt to use SBRT as an immune response trigger in combination with immunotherapy, no significant improvement in survival outcomes has been observed. The integration of new systemic agents with MDT will likely define a new scenario for the treatment of omUC. The review protocol was registered in PROSPERO, ID: CRD42024522381.

## 1. Introduction

Urothelial cancer (UC) is the most frequent neoplasm originating from the urinary system, accounting for approximately 90% of all bladder cancers (BCs), with a worldwide burden of more than half a million people affected and over 210,000 deaths in 2022 [1]. Nearly half of the new cases are diagnosed as confined muscle-invasive disease, while a minority of patients are metastatic at diagnosis. However, regardless of the treatments provided, the vast majority of confined UC patients develop metastases within two years of diagnosis [2]. Before presenting with widespread metastatic disease, some UC patients experience an intermediate state of disease characterized by the development of a limited number of metastases, so-called oligometastatic UC (omUC). A recent Delphi consensus agreed on the definition of this omUC status, which includes a limited number of a maximum of three metastases amenable to a metastasis-directed therapy (MDT) either with surgery or radiation [3].

In this scenario, the specific role of stereotactic body radiotherapy (SBRT), which delivers high radiation doses to limited volumes of disease with radical intent, has yet to be defined, but emerging evidence is increasingly demonstrating its potential [4].

In light of the unprecedented results of newly developed systemic treatments, such as antibody–drug conjugates (ADCs), in terms of survival, recently shown in the context of locally advanced or metastatic UC from the EV-302 trial [5], the scenario for omUC patients appears to be changing radically. Radiation therapy, for both symptom management and disease control, has been shown to be effective, safe, and affordable in the metastatic setting [6]. The recognition of omUC as a specific entity amenable to radical treatment opens up new scenarios in the context of prolonging survival and preserving quality of life.

The purpose of this paper is to systematically review the currently available evidence on the use of SBRT in omUC as an MDT strategy and to extract evidence on the main outcomes in terms of survival and local control benefit, as well as on potential biomarkers of treatment response.

## 2. Materials and Methods

This systematic review was conducted in accordance with the Preferred Reporting Items for Systematic Reviews and Meta-analyses guidelines [7] and registered in the international Prospective Register of Systematic Reviews (PROSPERO ID: CRD42024522381). A comprehensive search of PUBMED/Medline (NLM) as well as the ClinicalTrials.gov database was conducted by two researchers (A.A., D.G.B.). The literature search included retrospective and prospective studies on humans diagnosed with metastatic or advanced-stage UC and treated with SBRT, focusing on omUC patients.

The outcomes of interest were median overall survival (OS) and progression-free survival (PFS), local control (LC), and toxicity assessed with the Common Terminology Criteria for Adverse Events (CTCAE). Reports published in languages other than English were excluded. The search was restricted to the timeframe from 1 January 2006 to the search date (8 March 2024) to include studies with modern SBRT techniques starting from the first reported experiences with SBRT [8,9]. Prospective randomized or non-randomized studies as well as retrospective studies were included. Reviews, duplicates, studies with 5 patients or less, case reports, and reports without a clear SBRT description were excluded. The detailed search strategy and the results from all the database searches are available in the Supplementary Materials (Supplementary Table S1a–c, Search Strategy).

An interrater reliability test (Cohen’s Kappa) was carried out before screening all the citations found through the search [10]. Two authors (A.A., D.G.B.) independently screened the same sample of citations blinded to authors and journal titles. After the level of agreement was tested, A.A. and D.G.B. independently screened all titles and abstracts, still blinded to authors and journal titles, using an Excel workbook specifically designed for literature screening. Data were compiled into a single Excel workbook. Disagreements were discussed by the two screeners; if consensus could not be reached, a third person (T.Z.) familiar with the project provided final arbitration. The risk of bias was assessed for the selected studies using the Newcastle–Ottawa scale [11]. Scores of 7 or higher indicated studies with a low risk of bias. Final data were synthesized according to the SWiM (Synthesis Without Meta-analysis) checklist as a complementary extension of the PRISMA guidelines [12].

## 3. Results

**Figure 1:**
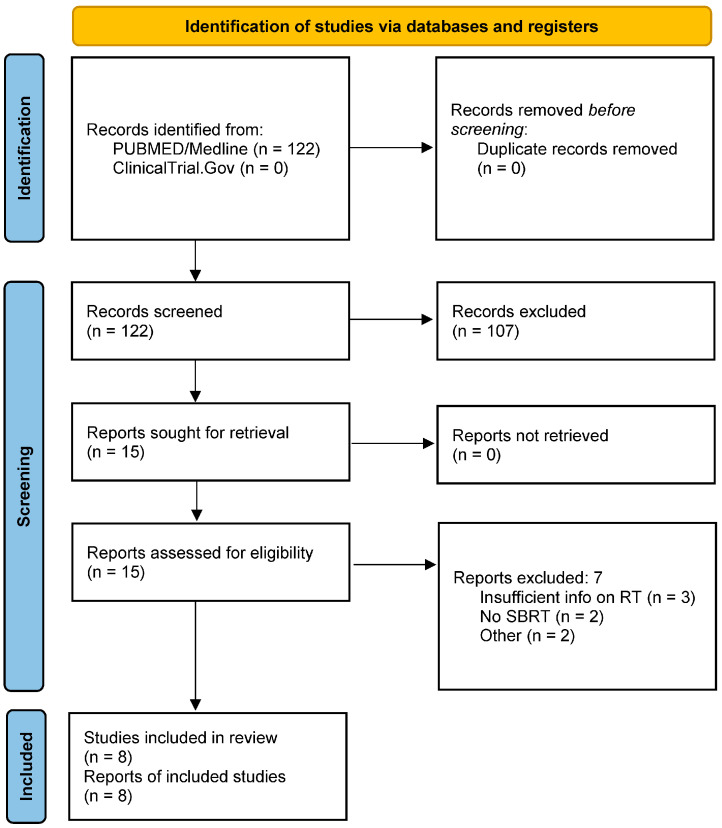
PRISMA flowchart.

The online database search identified 122 original works. The interrater reliability test based on random titles and abstracts showed high agreement among the investigators (Cohen’s K = 0.91, CI 0.8) (Supplementary Table S2. Cohen’s Kappa, level of agreement calculation). After the check for duplicates and the screening for titles and abstracts, 107 citations were excluded. Fifteen records were finally reviewed for the full text. Eight studies were ultimately included for the data extraction and analyzed in the present review, as illustrated in the PRISMA flowchart (Figure 1).

The exploration of heterogeneity among the studies underlined the lack of a robust definition of SBRT along with a slight difference in the definition of omUC disease, limiting the possibility to conduct a meta-analysis. Moreover, only two eligible studies showed a low risk of bias with a score of 7 or higher [13,14], while all the other studies showed a high [15,16,17,18,19], or in one case very high, risk of bias [20] (Supplementary Table S3: Risk of bias assessment).

The included studies involved a total of 357 patients, and 293 omUC patients were ultimately analyzed (Table 1).

Two studies were prospective (phase II or phase I trials) [18,19], while the others were retrospective multicenter (n = 2) [14,17] or single-institution (n = 4) [13,15,16,20] studies. The sample size ranged from 7 to 91 patients and included omUC patients with less than five metastatic lesions, or in two reports patients presenting up to three metastases [13,15,16,20]. 

Fifty-one patients presented synchronous oligometastases, while 242 were diagnosed with a metachronous oligometastatic disease. Only one study reported solely a metachronous omUC cohort [17]. In the metachronous setting, patients underwent mostly radical cystectomy [13] or first-line chemotherapy [14], with many studies not specifying previous systemic or local treatments.

Out of 293 omUC patients, 188 received SBRT as MDT with or without consolidative irradiation of the bladder. SBRT was delivered using modern RT techniques, with large variations in radiation doses and fractionation schedules among the studies (total dose: 24–60 Gy in 1–10 fractions). The biologically effective doses using an alpha/beta ratio = 10 Gy for tumor cells, namely BED10, ranged between 37.5 Gy and 151 Gy, with only one study clearly reporting a median BED10 of 78 Gy [17]. A BED10 < 60 Gy was delivered to the largest proportion of patients [14,15,18,19].

The median follow-up time ranged from 9 to 85 months. Reported clinical outcomes differed largely among studies, as summarized in Table 2.

In the multicenter analysis on omUC patients staged with either conventional or molecular imaging (presumably FDG-PET/CT), treated with a median SBRT BED10 dose of 78 Gy, Franzese et al. reported 2-year OS and PFS rates of 50% and 38%, respectively. Notably, the local control at 2 years was 88%. Nearly half of the patients (40%) were free from systemic treatment intensification at 2 years. No grade 3 adverse event was reported. In the univariate analysis, the number of chemotherapy lines before SBRT was correlated with lower local control (HR 2.62, *p* = 0.034) [17].

In a retrospective single-institution study, Miranda et al. [13] reported the highest OS rate (median OS of 51 months, 95% CI not calculable) among sixteen omUC patients previously treated with radical cystectomy (PFS of 8.2 months, 95% CI 1.4–5.5). In contrast, in a larger retrospective multicenter series, Aboudaram et al. [14] reported a lower median OS of 29 months and median PFS of 14 months among patients with omUC and with no progression following systemic therapy, similarly to the results observed by Franzese et al. in the metachronous omUC setting (median OS and PFS of 25 and 10 months, respectively) [17].

In the other studies included in the present analysis, the median OS ranged between 3.5 and 14 months. Notably, compared to other series, the two retrospective single-institution studies with the smallest sample size [15,20] reported lower median PFS values of 4.2 months and 2.9 months, respectively, suggesting that it is likely that the delivery of SBRT doses with BED10 values less than 60 Gy may have partially contributed to these disappointing results. Similarly, the use of sub-ablative SBRT doses of 24 Gy in three fractions (BED10 = 43 Gy) to one metastatic site with concomitant immunotherapy has been associated with a median PFS of 3.5 [18] and 4.4 months only, far below the median PFS reported in the other larger series using a higher BED10 SBRT dose [19]. These results are to be interpreted cautiously given the estimated high risk of bias for the current review question.

Concerning treatment-related toxicities, the highest rates of side effects were reported in a prospective multicenter clinical trial [19] testing a 24 Gy SBRT in three fractions with concurrent immunotherapy, where grade 3 CTCAE v5.0 toxicity was observed in 18% of the cases. All the other studies reported no severe (grade 3 or higher) acute or late adverse events, regardless of the association with concurrent systemic treatments or the delivered SBRT doses.

## 4. Discussion

The role of MDT in the management of omUC is a challenging open question. To date, robust evidence is missing. This is the first systematic review exploring the role of ablative SBRT in omUC according to the updated consensus definition proposed by a joint EAU-ESTRO-ESMO consensus [3]. We observed that the use of SBRT at ablative doses (BED_10_ ≥ 78 Gy) in the metachronous setting is associated with better outcomes compared to SBRT treatments with lower doses or systemic therapies. Although the evidence of survival benefit is not uniformly reported, these data have consistently shown a safe profile of SBRT treatments.

The use of SBRT combined with modern systemic therapies has been associated with promising synergistic effects in pre-clinical studies, but the concrete benefits in the clinical setting have to be proven [21]. The two prospective trials (a phase I and a randomized multicenter phase II trial) [18,19] investigating SBRT (24 Gy in three fractions) as MDT to 1–3 sites combined with immunotherapy both failed to demonstrate a significant gain in terms of OS or PFS, at the price of an increased rate of grade 3 toxicity. So far, expert groups have already promoted the pragmatic use of immunotherapy following ablative SBRT in the multidisciplinary management of UC, gathering opinions and real-world data to generate recommendations for routine application [22]. 

Regarding first-line systemic treatment, for decades, the main therapy relied on the use of platinum-based chemotherapy. In the last few years, the use of immunotherapy has shown promising results, and the recently published data on enfortumab–vedotin plus pembrolizumab have shown an unprecedented survival benefit over chemotherapy alone for untreated UC [5]. Considering the recent change in the first-line treatment of metastatic UC [23,24], several open questions may arise concerning the clinical applications of these new drugs in the real-world setting, specifically regarding the proper use and correct timing of local therapies such as SBRT.

Patient selection remains fundamental in selecting the optimal candidates for SBRT treatments. The use of baseline circulating tumor DNA (ctDNA), as explored in the phase 3 IMvigor010 trial evaluating adjuvant atezolizumab vs. placebo after cystectomy in muscle-invasive UC, may potentially be used as a useful tool for biomarker-driven patient selection for MDT strategies [25,26]. Although evidence for the application of biomarkers for SBRT in omUC is scarce, future studies focusing on omUC treatment should ideally implement novel biomarkers of disease burden and treatment response. 

18FDG-PET/CT could be a valuable tool to help select patients in the omUC setting [26]. Its role has been acknowledged in the updated EAU guidelines; however, evidence for its routinely use is still limited [24]. 

We acknowledge the limits of our review, mostly related to data consistency and lack of robustness, including heterogeneity in patient selection, including different definitions of omUC, treatment interventions (type of RT, SBRT dose, use of concurrent systemic treatments), and the reporting of the main outcomes. No meta-analysis was carried out, and the level of evidence to ultimately draw practical recommendations is low. 

Prospective data assessing the role of SBRT with or without the standard of care for UC will soon come from the NCT04724928 (EFFORTMIBC) study and a few other ongoing trials [4]. In light of emerging first-line treatments other than historical chemotherapy and technological improvements in radiotherapy for the safe delivery of MDT, new scenarios for the treatment of omUC patients are emerging. Studies with an appropriate and standardized design, taking into account the newly defined omUC entity, are needed to clarify these issues.

## 5. Conclusions

Despite the paucity and heterogeneity of the available literature, in the rapidly evolving landscape of omUC, SBRT delivered with ablative doses could be a safe and viable treatment strategy to improve the clinical outcomes of selected patients presenting with one to three metastatic lesions. The preference for SBRT as MDT should always be discussed in multidisciplinary boards to share the tailored decision for each clinical case. The use of novel systemic agents replacing standard platinum-based chemotherapy as a new standard of care will certainly define new therapeutic scenarios in which the role of SBRT as an MDT strategy will have to be determined in future studies.

## Figures and Tables

**Table 1:** Main characteristics of the selected studies.

Author, Year	Study Type	Population	Metastatic Setting	Sample Size	Intervention (Nr. of Patients)	BED10 Median, Gy (Range)	Comparison (Nr. of Patients)
	Group 1 (Patient sample N ≥ 15)						
Franzese, 2020 [17]	Retrospective, multicentric	omUC: ≤5 metastases	Synchronous 5%/ Metachronous 95%	61	SBRT +/− systemic treatment	78 Gy (37.5–151 Gy)	N/A
Aboudaram, 2023 [14]	Retrospective, multicentric	omUC: ≤5 metastases after 1st line CT	Synchronous/ Metachronous	91	1st line CT + RT (n = 51): -70% RT on bladder -SBRT (38 pts on 53 lesions)	62 Gy **	CT only (40)
Francolini, 2019 [16]	Retrospective, single-institution	omUC: ≤3 metastases	Metachronous	19	SBRT 60–18 Gy/8–1 fx +/− unspecified systemic treatment *	48 Gy (37.5–105 Gy)	N/A
Miranda, 2021 [13]	Retrospective, Single institution	omUC: ≤5 lesions at the time or after cystectomy	Synchronous 6%/ Metachronous 94%	52	MDT SBRT: 16 pts Palliative RT 60%/ consolidative RT 40%	N/A SBRT = >6 Gy/fr, 5 or less fractions	N/A
Spaas, 2023 [19]	Phase II trial, randomized multicentric,	Limited metastatic HNSCC, NSCLC melanoma, RCC, UC	Synchronous/ Metachronous	96 (UC:32 *)	SBRT 24 Gy/3 fx to 1–3 metastases and concurrent I.O. 2nd–3rd cycle (16 pts)	43.2 Gy	Standard of care: I.O. monotherapy (16 patients)
Sundhal, 2019 [18]	Phase I Trial	Metastatic UC with no brain involvement	N/A	18 *	SBRT 24 Gy/3 fx to 1 lesion concurrent to 2nd–3rd cycle I.O.	43.2 Gy	SBRT 24 Gy/3 fx to 1 lesion prior to 1st cycle I.O.
	Group 2 (Patient sample N < 15)						
Augugliaro, 2018 [15]	Retrospective single-institution	omUC: ≤5 metastases (node, bone, or lung)	N/A	13	SBRT 36–20 Gy/5 fx (3–10 fx)	35.7 Gy (28–60 Gy)	N/A
Leonetti, 2018 [20]	Retrospective single-institution	omUC: ≤3 metastases	Synchronous 14%/ Metachronous 86%	7	SBRT 40–25 Gy/5 fx +/− systemic treatment (CBCDA or CDDP/Gem)	48 Gy (37.5–72 Gy)	N/A

* The exact number of metastases for each patient is not clearly reported. ** Reported as EQD2: 53 Gy (45–132 Gy). Abbreviations: UC = urothelial cancer, omUC: oligometastatic urothelial cancer; UUT = upper urinary tract; (I) = intervention; (C) = comparison; ORR = objective response rate; LCR = local control rate; LPFI = local progression-free interval; OS = overall Survival; PFS = progression-free survival; DLT = defined as any grade 3–5 metabolic or hematological toxicity or any grade 3–5 non-hematological toxicity that was (probably or possibly) related to SBRT; N/A = not applicable; HNSCC = head and neck squamous cell carcinoma; NSCLC = non-small-cell lung cancer; CT = chemotherapy; I.O. = immunooncology agent(s); BED = biologically effective dose (α/β = 10 Gy); EQD2 = 2 Gy fraction-equivalent dose.; SBRT = stereotactic body radiation therapy; CBCDA = carboplatin; CDDP = cisplatin; Gy = gray; Fx = radiotherapy fraction.

**Table 2:** Outcomes of the selected studies.

Author, Year [Ref.]	Median FU Time, Range (Months)	Outcomes and Side Effects				Main Remarks
		Local Control	Median PFS (Months)	Median OS (Months)	Toxicity (CTCAE v5.0)	
Group 1 (Patient sample N > 15)						
Franzese, 2020 [17]	17.2 (3–91)	1y-LC: 92%, 2y-LC: 88%	10 1yPFS = 47% 2yPFS = 38%	25.6 1yOS = 78.9% 2yOS = 50.7%	Acute/late: G > 3: 0/0	2yFFIT: 40%
Aboudaram, 2023 [14]	85.9 (36–101)	N/A	14.8 [I] vs. 9.7 [C] *p* = 0.08	29.7 [I] vs. 19.7 [C] *p* = 0.074	Acute/late: G > 3: 0/0	Whole population: OS: 21.7 M PFS: 11.1 M
Francolini, 2019 [16]	11.5 (1–44)	1y-LC: 68%	5.6	13.8	Acute/lateG > 3: 0/0	ORR:40%
Miranda, 2021 [13]	26.6 (18.1–39.5)	1yLC = 72%	8 Rates(%): 2yPFS = 19	51 Rates(%): 2yOS = 60	Acute/late: G ≥ 3: 4%	
Spaas, 2023 [19]	12.5 (0.7–46.2)	1yLC = 76% § iCR = 16% §	4.4 [I] vs. 2.8 [C] *p* = 0.82 §	14.3 [I] vs. 11 [C] *p* = 0.47 §	G ≥ 3:18% no difference between arms	Absolute lymphocyte count changes: 3.0%[C] vs. −13.6%[I] *p* = 0.006
Sundhal, 2019 [18]	9 (4–14)	LCR: CR: <30% [C] vs. 50% [I]	3.5 [I] vs. 3.3 [C] *p* = N/A	12.1 [C] vs. 3.5 [I] *p* = N/A	Arm I = G1–2 vs. Arm C = G1 Overall G > 3 = 0	ORR = 0[C] vs. 44%[I] 3PR, 1CR SD 50% in both arms
Group 2 (Patient sample N < 15)						
Augugliaro, 2018 [15]	25 (3–43)	4 months LC: 57% (PR,CR,SD)	4.2	N/A	G > 2 = 0	Local failure 9 pts: 6 pts in field + distant PD
Leonetti, 2018 [20]	Unclear (5–16)	1yLC: 100% (PR,CR,SD)	2.9	14	G > 1 = 0	LPFI > with 40 Gy/5 fx than with 25 Gy/5 fx

§ cumulative data from 99 patients with different primary histology, with no specific info for the 32 UC. Abbreviations: UC = urothelial cancer; omUC: oligometastatic urothelial cancer; UUT = upper urinary tract; [I] = intervention; [C] = comparison; ORR = objective response rate; LCR = local control Rate; iCR = iRECIST-defined complete response; LPFI = local progression-free interval; OS = overall survival; PFS = progression-free survival; DLT = defined as any grade 3–5 metabolic or hematological toxicity or any grade 3–5 non-hematological toxicity that was (probably or possibly) related to SBRT; N/A = not applicable; Cht = chemotherapy; I.O. = immunooncology agent(s); BED = biologically effective dose (α/β = 10 Gy); EQD2 = 2 Gy fraction-equivalent dose.; SBRT = stereotactic body radiation therapy; CBCDA = carboplatin; CDDP = cisplatin; Gy = gray; Fr = radiotherapy fraction; FFIT = free from intensification of treatment(s).

## Data Availability

The data presented in this study are available in this article and Supplementary Materials.

## Supplementary Materials

The following supporting information can be downloaded at https://www.mdpi.com/article/10.3390/cancers16183201/s1: Supplementary Table S1a: Search strategy in PUBMED/Medline (NML); Supplementary Table S1b: search strategy in Clinicaltrials.gov; Supplementary Table S1c: database search resumed; Supplementary Table S2: Cohen’s Kappa, level of agreement calculation; Supplementary Table S3: risk of bias assessment.

## References

[1] Bray F., Laversanne M., Sung H., Ferlay J., Siegel R.L., Soerjomataram I., Jemal A. (2024). Global cancer statistics 2022: GLOBOCAN estimates of incidence and mortality worldwide for 36 cancers in 185 countries. CA Cancer J. Clin..

[2] Bamias A., Stenzl A., Zagouri F., Andrikopoulou A., Hoskin P. (2023). Defining Oligometastatic Bladder Cancer: A Systematic Review. Eur. Urol. Open Sci..

[3] Bamias A., Stenzl A., Brown S.L., Albiges L., Babjuk M., Birtle A., Briganti A., Burger M., Choudhury A., Colecchia M. (2023). Definition and Diagnosis of Oligometastatic Bladder Cancer: A Delphi Consensus Study Endorsed by the European Association of Urology, European Society for Radiotherapy and Oncology, and European Society of Medical Oncology Genitourinary Faculty. Eur. Urol..

[4] Huynh M.A., Tang C., Siva S., Berlin A., Hannan R., Warner A., Koontz B., De Meeleer G., Palma D., Ost P. (2023). Review of Prospective Trials Assessing the Role of Stereotactic Body Radiation Therapy for Metastasis-directed Treatment in Oligometastatic Genitourinary Cancers. Eur. Urol. Oncol..

[5] Powles T., Valderrama B.P., Gupta S., Bedke J., Kikuchi E., Hoffman-Censits J., Iyer G., Vulsteke C., Park S.H., Shin S.J. (2024). Enfortumab Vedotin and Pembrolizumab in Untreated Advanced Urothelial Cancer. N. Engl. J. Med..

[6] Ashley S., Choudhury A., Hoskin P., Song Y., Maitre P. (2024). Radiotherapy in metastatic bladder cancer. World J. Urol..

[7] Page M.J., McKenzie J.E., Bossuyt P.M., Boutron I., Hoffmann T.C., Mulrow C.D., Shamseer L., Tetzlaff J.M., Akl E.A., Brennan S.E. (2021). The PRISMA 2020 statement: An updated guideline for reporting systematic reviews. BMJ.

[8] Nagata Y., Nagata Y. (2015). Introduction and History of Stereotactic Body Radiation Therapy (SBRT). Stereotactic Body Radiation Therapy: Principles and Practices.

[9] Kavanagh B.D., McGarry R.C., Timmerman R.D. (2006). Extracranial radiosurgery (stereotactic body radiation therapy) for oligometastases. Semin. Radiat. Oncol..

[10] McHugh M.L. (2012). Interrater reliability: The kappa statistic. Biochem. Med..

[11] Stang A. (2010). Critical evaluation of the Newcastle-Ottawa scale for the assessment of the quality of nonrandomized studies in meta-analyses. Eur. J. Epidemiol..

[12] Campbell M., McKenzie J.E., Sowden A., Katikireddi S.V., Brennan S.E., Ellis S., Hartmann-Boyce J., Ryan R., Shepperd S., Thomas J. (2020). Synthesis without meta-analysis (SWiM) in systematic reviews: Reporting guideline. BMJ.

[13] Miranda A.F., Howard J.M., McLaughlin M., Meng X., Clinton T., Sanli O., Garant A., Bagrodia A., Margulis V., Lotan Y. (2021). Metastasis-directed radiation therapy after radical cystectomy for bladder cancer. Urol. Oncol..

[14] Aboudaram A., Chaltiel L., Pouessel D., Graff-Cailleaud P., Benziane-Ouaritini N., Sargos P., Schick U., Crehange G., Cohen-Jonathan Moyal E., Chevreau C. (2023). Consolidative Radiotherapy for Metastatic Urothelial Bladder Cancer Patients with No Progression and with No More than Five Residual Metastatic Lesions Following First-Line Systemic Therapy: A Retrospective Analysis. Cancers.

[15] Augugliaro M., Marvaso G., Ciardo D., Zerini D., Riva G., Rondi E., Vigorito S., Comi S., De Cobelli O., Orecchia R. (2019). Recurrent oligometastatic transitional cell bladder carcinoma: Is there room for radiotherapy?. Neoplasma.

[16] Francolini G., Desideri I., Detti B., Di Cataldo V., Masi L., Caramia G., Visani L., Terziani F., Muntoni C., Lo Russo M. (2019). Stereotactic radiotherapy in oligoprogressive and oligorecurrent urothelial cancer patients: A retrospective experience. Cancer Treat. Res. Commun..

[17] Franzese C., Francolini G., Nicosia L., Alongi F., Livi L., Scorsetti M. (2021). Stereotactic Body Radiation Therapy in the Management of Oligometastatic and Oligoprogressive Bladder Cancer and Other Urothelial Malignancies. Clin. Oncol..

[18] Sundahl N., Vandekerkhove G., Decaestecker K., Meireson A., De Visschere P., Fonteyne V., De Maeseneer D., Reynders D., Goetghebeur E., Van Dorpe J. (2019). Randomized Phase 1 Trial of Pembrolizumab with Sequential Versus Concomitant Stereotactic Body Radiotherapy in Metastatic Urothelial Carcinoma. Eur. Urol..

[19] Spaas M., Sundahl N., Kruse V., Rottey S., De Maeseneer D., Duprez F., Lievens Y., Surmont V., Brochez L., Reynders D. (2023). Checkpoint Inhibitors in Combination with Stereotactic Body Radiotherapy in Patients with Advanced Solid Tumors: The CHEERS Phase 2 Randomized Clinical Trial. JAMA Oncol..

[20] Leonetti A., D’Abbiero N., Baldari G., Andreani S., Ruffini L., Viansone A.A., Buti S. (2018). Radiotherapy for the treatment of distant nodes metastases from oligometastatic urothelial cancer: A retrospective case series. Int. J. Urol..

[21] Wilkins A., Ost P., Sundahl N. (2021). Is There a Benefit of Combining Immunotherapy and Radiotherapy in Bladder Cancer?. Clin. Oncol..

[22] Gonzalez-Del-Alba A., Conde-Moreno A.J., Garcia Vicente A.M., Gonzalez-Peramato P., Linares-Espinos E., Climent M.A., The Sogug Multidisciplinary Working G. (2022). Management of Patients with Metastatic Bladder Cancer in the Real-World Setting from the Multidisciplinary Team: Current Opinion of the SOGUG Multidisciplinary Working Group. Cancers.

[23] Powles T., Bellmunt J., Comperat E., De Santis M., Huddart R., Loriot Y., Necchi A., Valderrama B.P., Ravaud A., Shariat S.F. (2024). ESMO Clinical Practice Guideline interim update on first-line therapy in advanced urothelial carcinoma. Ann. Oncol..

[24] Witjes J.A., Bruins H.M., Carrión A., Cathomas R., Compérat E.M., Efstathiou J.A., Fietkau R., Gakis G., van der Heijden A.G., Lorch A. EAU Guidelines on Muscle-invasive and Metastatic Bladder Cancer. https://d56bochluxqnz.cloudfront.net/documents/full-guideline/EAU-Guidelines-on-Muscle-Invasive-and-Metastatic-Bladder-Cancer-2024.pdf.

[25] Powles T., Assaf Z.J., Degaonkar V., Grivas P., Hussain M., Oudard S., Gschwend J.E., Albers P., Castellano D., Nishiyama H. (2024). Updated Overall Survival by Circulating Tumor DNA Status from the Phase 3 IMvigor010 Trial: Adjuvant Atezolizumab Versus Observation in Muscle-invasive Urothelial Carcinoma. Eur. Urol..

[26] Oldan J.D., Schroeder J.A., Hoffman-Censits J., Rathmell W.K., Milowsky M.I., Solnes L.B., Nimmagadda S., Gorin M.A., Khandani A.H., Rowe S.P. (2024). PET/Computed Tomography Transformation of Oncology: Kidney and Urinary Tract Cancers. PET Clin..

