# Supplementary material for: Oligometastatic Urothelial Cancer and Stereotactic Body Radiotherapy: A Systematic Review and an Updated Insight of Current Evidence and Future Directions
Source: Cancers (Basel). 2024 Sep 20;16(18):3201. doi: 10.3390/cancers16183201 (PMC11430162; doi:10.3390/cancers16183201)
Supplement: Supplementary file 1 [file cancers-16-03201-s001.zip › cancers-3197515-supplementary.pdf]

Supplementary Table S1a. Search Strategy PUBMED/Medline (NML).

Search Terms	
#1 (Urothelial Cancer)	((bladder cancer) OR (urothelial cancer) OR (upper urinary tract cancer)) OR (urethral cancer) OR (urothelial bladder cancer))
#2 (Oligometastatic)	(((oligometastatic) OR (metastatic)) OR (advanced stage))
#3 (Stereotactic body radiation therapy)	((metastasis-directed radiotherapy) OR (stereotactic body radiotherapy)) OR (sbrt) OR (ablative radiotherapy) OR (consolidative radiotherapy) OR (metastases directed radiotherapy))
#1 AND #2 AND #3	

Language: English only

Filters:

- Year of publication from 2006 to date of search

Supplementary Table S1b. Search Strategy Clinicaltrials.gov.

**Provider/Interface**	NLM
**Database**	Clinicaltrials.gov
**Date searched**	08/03/2024
**Database update**	07/03/2024
**Search developer(s)**	AA, DGB
**English only? (default is "Yes")**	Yes
**Date range**	No limit by date
**Publication types included**	Clinical trials
**Search filter source**	No search filter used
**Items found**	0
**# of deleted duplicates from columns F-G-H**	0
**Unique items found**	0
**Name of saved search: SBRT and omUC**	
**Search terms**	
Condition or Disease	Urothelial cancer
Intervention/ Treatment	Stereotactic Body Radiotherapy
Other	Oligometastatic disease

Supplementary Table S1c. Database Search resumed.

Table ID	Provider/Interface	Database	Date searched	Database update	Searcher(s)	English only?	Date restriction	Pub types excluded	Search filter source	Original # of Items found	# of deleted duplicates	# of Unique items found
1a	National Library of Medicine	PubMed	March 8, 2024	March 8, 2024	AA, DGB	Yes	from 2006	No limit by publication type	Humans	122	0	122
1b	NLM	Clinicaltrials.gov	March 8, 2024	March 8, 2024	AA, DGB	Yes	No limit by date	Clinicaltrials	No search filter used	0	0	0

	Original # of Items found	# of deleted duplicates	# of Unique items found
**Total**	122	0	122

Supplementary Table S2. Cohen's KAPPA, Level of agreement calculation.

$$\kappa = \frac{\Pr(a) - \Pr(e)}{1 - \Pr(e)}$$

					Observed Agreement					
	Go to full text review	Exclude	# screened		Both S1 and S2 said "Go to full text review" to same records or "Exclude" to same records	Pr(a) = (# both said "Go to full text review" and "exclude")/(total number screened), i.e. raw agreement	Raw % of Go to full text review	Raw % of Exclude	"Pr(e)" = Probability of random agreement = ((Raw % of S1 Go to full text review")*(Raw % of S2 "Go to full text review")) + ((Raw % of S1 No's)*(Raw % of S2 No's))	Cohen's Kappa
Screener 1 (S1)	25	41	66	Compiled	63	0.95	0.38	0.62	0.52	0.91
Screener 2 (S2)	28	38	66				0.42	0.58		
		Did you screen the same number?	yes	If you do not show the same number screened, you cannot accurately calculate the Cohen's Kappa.						

Standard error of Kappa	Confidence interval low end	Confidence interval high end
0.053235016	0.801283774	1.009965035

		Screener 2	
		Go to full text review	Exclude
Screener 1	Go to full text review	25	0
	Exclude	3	38

Raw % that each said Exclude or Go to full text review			Probability of random agreement	
	Go to full text review	Exclude		
Screener 1	0.38	0.62	0.52	
Screener 2	0.42	0.58		

$$SE_{\kappa} = \sqrt{\frac{p\,(1-p)}{n\,(1-p_e)^2}}$$

Supplementary Table S3. Risk of bias assessment.

Study Author, year	Newcastle-Ottawa Scale Items: Selection – Representativeness of the Exposed Cohort	Selection – Selection of the Non-Exposed Cohort	Selection – Ascertainment of Exposure	Selection – Demonstration That Outcome of Interest Was Not Present at Start of	Comparability – 1)Comparability of Cohorts on the Basis of the Design or Analysis		Outcome – Assessment of Outcome	Outcome – Follow-Up Long Enough for Outcomes to	Outcome – Adequacy of Follow Up of Cohorts	Sum n. of Stars	
**Aboudaram, 2023**	b) *	a) *	a) *	a)*	a, b)**	omUC <5met, I line chem	b)*	a)*	b)*	9/9	Low RoB
**Miranda, 2021**	b) *	b)	a) *	a)*	*	omUC <5met, after cyste	b)*	a)*	b)*	7/9	Low RoB
**Franzese, 2020**	b) *	c)	a) *	a)*	n.a.		b)*	a)*	b)*	6/9	High RoB
**Francolini, 2019**	b) *	c)	a) *	a)*	n.a.		b)*	a)*	b)*	6/9	High RoB
**Augugliaro, 2018**	b) *	c)	a) *	a)*	n.a.		b)*	a)*	b)*	6/9	High RoB
**Leonetti, 2018**	b) *	c)	a) *	a)*	n.a.		b)*	b)	c)	4/9	Very high Rob

Confounding Domains checked ad hoc for prospective Phase I or Phase II Trial (Inspired by NOS scale and ROBINS-I)	Patient selection		Intervention				Outcome		
**Spaas, 2023**	Heterogeneous sample of patients, according to: - Different histology (HNSCC, NSCLC, Melanoma, RCC, UC)	High total number of patients recruited, however, only 32 out 96 patients were omUC, and only 16 received SBRT.	Sub-ablative Radiation doses for SBRT plus concurrent I.O. The decision rationale for the radiation dose delivered was mentioned	Standardized intervention: Radiation and I.O. vs I.O. alone (standard of care)	SBRT with 1 to 3 metastatic sites targeted, thus not all patients received SBRT as MDT to all the metastases.	Overall balanced study to assess no benefits from the Intervention vs comparison. Not exhaustive data regarding SBRT and omUC specificaly.	Follow-up time and assessment is appropriate with the evaluated outcomes	Measure of outcome standardised, it seem to be appropriate	High RoB
**Sundhal, 2019**	Quite homogeneous sample	Unclear number of metastasis, no brain mets	Sub-ablative Radiation doses for SBRT plus concurrent I.O. The decision rationale for the radiation dose delivered was mentioned	standardized intervention: Radiation and I.O. either concurrent or before 1°cycle I.O.	SBRT with 1 to 3 metastatic sites targeted, thus not all patients received SBRT as MDT to all the metastases.	Balanced study between the Intervention vs comparison arms. Not exhaustive data regarding SBRT and omUC specifically.	follow-up time and assessment seems appropriate with the study outcomes , however it is shorter than f-up time of	measure of outcome seem to be appropriate for the study aims, not fitting exhaustively the review question	High RoB

Scoring: 7-9 =Low Rob (Green) | 4-6= High Rob (yellow) | 0-3= Very High Rob (red)
